# Ischemic stroke associated with adenomyosis-related abnormal uterine bleeding: a systematic review of management and outcomes

**DOI:** 10.3389/fneur.2025.1698533

**Published:** 2025-12-04

**Authors:** Limei Zheng, Hong Zhang, Wei Fang, Jianhua Yang

**Affiliations:** 1 Assisted Reproduction Unit, Department of Obstetrics and Gynecology, Sir Run Run Shaw Hospital, School of Medicine, Zhejiang University, Hangzhou, China; 2 Zhejiang Provincial Clinical Research Center for Obstetrics and Gynecology, Hangzhou, China; 3 Zhejiang Key Laboratory of Precise Protection and Promotion of Fertility, Zhoushan, China; 4 Department of Obstetrics and Gynaecology, People’s Hospital of Putuo District, Zhoushan, China; 5 Department of Neurology, Hangzhou Hospital of Traditional Chinese Medicine Hospital Affiliated to Zhejiang Chinese Medical University, Hangzhou, Zhejiang, China

**Keywords:** adenomyosis, abnormal uterine bleeding, cerebral infarction, conservative treatment, operation treatment

## Abstract

**Background:**

Adenomyosis is frequently associated with abnormal uterine bleeding (AUB) and anemia. In the setting of arterial stenosis or hypercoagulability, AUB may precipitate ischemic stroke. However, the optimal management strategy remains unclear.

**Methods:**

In accordance with PRISMA 2020, we systematically searched PubMed, Web of Science, CNKI, and Wanfang from inception to November 20, 2024, without language restrictions. Eligible studies were human case reports or case series that described adenomyosis-associated ischemic stroke with extractable data on management and outcomes. Data extraction was performed independently by two reviewers, and risk of bias was assessed using the JBI tool. A descriptive synthesis was conducted, and Fisher’s exact tests were applied where appropriate.

**Results:**

Eighteen studies involving 24 patients fulfilled the inclusion criteria. Among these patients, 66.7% experienced stroke onset during menstruation, 85.7% presented with anemia, and more than 95% showed elevated CA125 and D-dimer levels. Stroke recurrence occurred in 55.6% of patients who received conservative management, compared with 0% of those who underwent hysterectomy (Fisher’s exact p ≈ 0.005). In menstruation-related cases managed conservatively, the recurrence rate reached 83.3%.

**Conclusion:**

Current evidence indicates that rapid uterine hemostasis may facilitate timely initiation of antithrombotic therapy and thereby reduce recurrence risk in adenomyosis-associated stroke. Surgical management, particularly hysterectomy, appears more effective than conservative therapy in carefully selected high-risk patients. Larger prospective studies are needed to confirm these findings and to refine management strategies.

## 1 Introduction

Ischemic stroke, resulting from the interruption of cerebral blood flow, remains one of the leading causes of morbidity and mortality worldwide. Established risk factors include atherosclerosis, thrombosis, hypertension, metabolic disorders, cigarette smoking, and alcohol consumption. Although stroke has been extensively investigated in the context of cardiovascular and metabolic diseases, its potential association with gynecological conditions—particularly abnormal uterine bleeding (AUB)—has received limited attention.

Adenomyosis is a benign gynecological disorder characterized by the presence of ectopic endometrial glands and stroma within the myometrium. Clinically, it frequently manifests as heavy menstrual bleeding, dysmenorrhea, and iron-deficiency anemia. In patients with coexisting cerebrovascular stenosis or a hypercoagulable state, significant uterine bleeding may exacerbate cerebral hypoperfusion or activate thrombotic pathways, thereby increasing the risk of ischemic stroke. However, the pathophysiological link between adenomyosis and stroke remains poorly understood and is rarely explored in the literature.

Currently, there is no consensus or standardized protocol for managing patients with adenomyosis-associated AUB who develop ischemic stroke. Clinical decision-making in such cases is particularly challenging due to conflicting therapeutic priorities: the urgent need for hemostasis versus the risk of withholding antithrombotic therapy. To date, only a limited number of case reports have documented such presentations, and little is known about the comparative efficacy of medical versus surgical interventions in this population.

Given the rarity but clinical importance of this intersection between gynecology and neurology, we conducted a systematic review of published cases of ischemic stroke associated with adenomyosis-related AUB. Our objectives were to summarize the clinical and laboratory characteristics of reported patients, and evaluate treatment approaches and outcomes, with particular emphasis on the relative risks of recurrence after conservative versus surgical interventions.

## 2 Methods

### 2.1 Search strategy

Following the PRISMA 2020 guidelines, we systematically searched PubMed, Web of Science, CNKI (China National Knowledge Infrastructure), and Wanfang Data from database inception through November 20, 2024, without language restrictions. Database-specific strategies, including Boolean operators and English/Chinese search terms, are provided in Supplementary File S1.

### 2.2 Eligibility criteria

#### 2.2.1 Inclusion criteria

Studies were eligible if they met all of the following conditions:

(a) Female patients with imaging-confirmed ischemic stroke;
(b) Clinical or imaging-based diagnosis of adenomyosis;
(c) Reported at least one of the following parameters: hemoglobin (Hb), CA125, or D-dimer levels; or provided a clearly documented treatment approach and outcomes.

#### 2.2.2 Exclusion criteria

Studies were excluded if they met any of the following conditions:

(a) Malignancy-associated stroke (e.g., endometrial carcinoma);
(b) Non-ischemic stroke;
(c) Overlapping populations or duplicate reports.

### 2.3 Study selection

**Figure 1:**
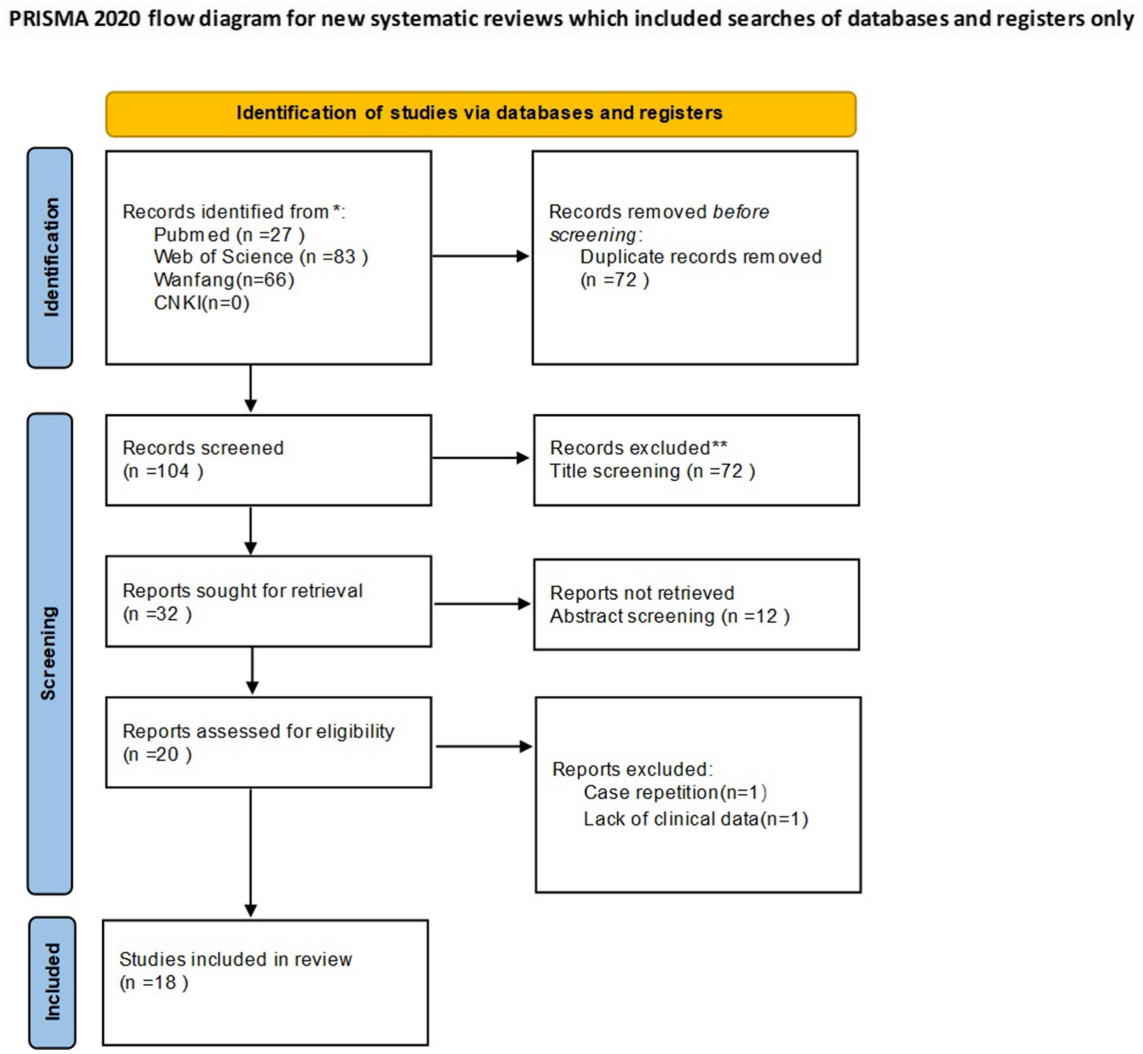
PRISMA 2020 flow diagram of the literature search and selection process.

Two reviewers independently screened all titles and abstracts, followed by full-text assessments of potentially eligible studies. Discrepancies were resolved through discussion with a third reviewer. The study selection process is presented in the PRISMA flow diagram (Figure 1).

### 2.4 Risk of bias

Risk of bias was evaluated using the JBI critical appraisal checklists for case reports and case series. Items assessed included patient demographics, clinical history, diagnostic ascertainment, clarity of interventions, adequacy of follow-up, and outcome reporting. Risk-of-bias summaries are shown in Supplementary Table S2.

### 2.5 Data synthesis and statistical analysis

Proportions were summarized as numerators and denominators. Where appropriate, Fisher’s exact tests were used to compare recurrence rates between conservative and surgical management groups, with two-sided significance set at *α* = 0.05. Analyses were descriptive given the small sample size and heterogeneity of included studies.

## 3 Results

### 3.1 Study selection

The database search identified 176 records. After removal of duplicates, 104 records remained. Following title and abstract screening, 20 full texts were assessed, and 18 studies met the inclusion criteria. The selection process is summarized in Figure 1.

### 3.2 Patient characteristics

Patients with adenomyosis-associated ischemic stroke typically presented to neurology departments with acute neurological symptoms, including motor deficits, speech disturbances, altered consciousness, or visual impairments. All included case reports originated from Asia. Across 24 patients, the mean age was 45.2 ± 5.6 years. Stroke onset occurred during menstruation in 66.7% of patients, anemia was reported in 85.7%, and more than 95% demonstrated elevated CA125 and D-dimer levels. Half of the patients (50%) exhibited evidence of extracranial thrombosis, including nonbacterial thrombotic endocarditis (NBTE) and thromboses in the kidneys, lungs, spleen, or peripheral vessels. Detailed individual characteristics are provided in Supplementary Table S3 and Table 1; (1–18).

**Table 1:** Summary of clinical characteristics in adenomyosis patients with cerebral infarction.

Patient demographics and clinical features		
Variable	Category	Cases, n/N (%)
Initial consultation		
	Neurology	24/24 (100%)
	Gynecology	0/24 (0%)
Country		
	Japan	16/24 (66.7%)
	China	6/24 (25%)
	Korea	2/24 (8.3%)
Age distribution		
	31–40 years	4/24 (16.7%)
	41–50 years	19/24 (79.2%)
	51–60 years	1/24 (4.1%)
Neurological symptoms		
	Limb symptoms*	14/24 (58.3%)
	Aphasia	6/24 (25%)
	Impaired consciousness	5/24 (20.8%)
	Hemianopsia	2/24 (8.3%)
	Epilepsy	1/24 (4.2%)
Gynecological symptoms		
	Onset during menstruation	14/21 (66.7%)
	Onset during non-menstrual period	7/21 (33.3%)
Laboratory findings		
CA-125	Elevated (> = 35 U/mL)	22/23 (95.6%)
	Normal	1/23 (4.3%)
D-dimer	Elevated (>0.5 μg/mL)	22/23 (95.6%)
	Normal	1/23 (4.3%)
Hemoglobin	Normal (≥12 g/dL)	3/21 (14.3%)
	Mild anemia (9–11.9 g/dL)	7/21 (33.3%)
	Moderate anemia (6–8.9 g/dL)	9/21 (42.9%)
	Severe anemia (<6 g/dL)	2/21 (9.5%)
Other thrombotic events		
	NBTE	5/24 (20.8%)
	Kidney	4/24 (16.7%)
	Extremities	2/24 (8.3%)
	Extracranial vessels	2/24 (8.3%)
	Spleen	1/24 (4.2%)
	Lung	1/24 (4.2%)
	Fingers	1/24 (4.2%)
Treatment for cerebral infarction		
	Medical therapy	20/21 (95.2%)
	Vascular reconstruction	1/21 (4.8%)
	Thrombectomy	1/21 (4.8%)
Treatment for adenomyosis		
Medical therapy	GnRH agonist	9/10 (90%)
	Progesterone	1/10 (10%)
Surgical therapy	Total hysterectomy	13/22 (59.1%)
Disease recurrence		
	Recurrence after conservative treatment	5/9 (55.6%)
	Recurrence after surgery	0/13 (0%)
	*p* = 0.005	

CA-125, Cancer Antigen 125. GnRH, Gonadotropin-Releasing Hormone. NBTE, Nonbacterial Thrombotic Endocarditis. *Limb symptoms include weakness, hemiparesis, hemiplegia, and ataxia. **Chi-square test was performed for comparison of recurrence rates. ***Anemia defined as hemoglobin <120 g/L. Data not available for all cases in certain categories, explaining variations in denominators. Statistical significance set at *p* < 0.05.

### 3.3 Treatment and outcomes

For acute ischemic stroke, 95.2% of patients received antithrombotic therapy (either antiplatelet agents or anticoagulation). A minority underwent vascular interventions: 4.8% received endovascular revascularization and 4.8% underwent mechanical thrombectomy.

Regarding adenomyosis management, 10 patients received conservative medical therapy; of these, 90% were treated with gonadotropin-releasing hormone (GnRH) agonists and 10% with medroxyprogesterone. Among the 10 patients with available follow-up data, the recurrence rate under conservative treatment was 55.6%. In contrast, 13 patients (59.1%) underwent surgical intervention of hysterectomy, and none experienced recurrence. The recurrence rate was significantly higher in the conservative group compared with the surgical group (*p* < 0.05; Table 1). Subgroup analysis (Table 2) indicated that recurrence was strongly associated with menstruation-related symptom onset in conservatively managed patients, although statistical significance was not reached due to limited sample size.

**Table 2:** Subgroup analysis of recurrence rates following conservative treatment.

Variable	Conservative treatment recurrence	*p*-value*
Onset timing		
During menstruation	5/6 (83.3%)	0.107
Non-menstrual period	0/2 (0%)	
Anemia status		
With anemia	4/5 (80%)	1
Without anemia	1/1 (100%)	

Statistical Analysis: *Chi-square test or Fisher’s exact test (when expected cell count <5). Statistical significance set at *p* < 0.05.

### 3.4 Risk of bias

Common methodological issues included reliance on partial gynecological clinical data, missing diagnostic or laboratory information, incomplete follow-up, and inherent limitations of case report designs. Nevertheless, the main findings remained consistent across studies.

## 4 Discussion

Adenomyosis-related abnormal uterine bleeding (AUB) has emerged as a rare but clinically important trigger of ischemic stroke. At present, no standardized guidelines exist for managing such cases, and physicians often face a therapeutic dilemma between initiating antithrombotic therapy and achieving hemostasis. This systematic review provides insights into the potential pathophysiological link between adenomyosis, coagulation activation, and cerebrovascular ischemia, and highlights the relative risks and benefits of conservative versus surgical interventions. Collectively, the evidence suggests that achieving definitive uterine hemostasis may reduce recurrence risk and facilitate safer secondary stroke prevention.

### 4.1 Pathophysiology of stroke in adenomyosis patients

Although adenomyosis is a benign gynecological condition, it may contribute to an increased risk of stroke through mechanisms beyond traditional stroke etiologies. Prior studies have highlighted the role of hypercoagulable states in ischemic stroke patients without conventional stroke mechanisms (CSMs) such as large artery atherosclerosis, small vessel disease, cardioembolism or other determined etiology (19, 20). In adenomyosis, elevated serum markers such as CA125, CA19-9, and D-dimer suggest the presence of a mucin-related hypercoagulable state. Some researchers propose that benign mucin-producing tumors may promote thrombosis through the mutual activation of adhesion signals between neutrophils and platelets (2, 8, 9, 21, 22).

Additional mechanisms may include the high expression of tissue factor (TF) in endometrial tissue, the release of microparticles, and the chronic inflammation and bleeding induced by ectopic endometrial tissue within the myometrium, all of which can activate the coagulation cascade and trigger thromboinflammatory responses (23–26). Anemia and acute blood loss due to heavy menstrual bleeding and persistent abnormal vaginal bleeding further amplify thrombotic risk. Hemodynamic disturbances, including compensatory blood flow and increased turbulence, along with enhanced platelet adhesion and reduced fibrinolytic activity during acute bleeding episodes, may also contribute to thrombosis formation (27, 28).

In this review, most patients were premenopausal women (except for one 59-year-old receiving hormone replacement therapy). Of these, 66.7% experienced stroke onset during menstruation, over 95% had elevated CA125 and D-dimer levels, 85.7% had anemia, and 50% showed extracranial thrombosis. These findings support the proposed underlying mechanisms. While anemia and a hypercoagulable state were common findings among the patients reviewed, not all exhibited these conditions. Notably, one patient with normal levels of CA125 and D-dimer developed ischemic stroke despite acute bleeding and cerebral vascular stenosis. This suggests that other factors, such as vascular stenosis or undiagnosed coagulation disorders (e.g., acute bleeding), may also contribute to the occurrence of stroke. The close temporal relationship between the patient’s abnormal uterine bleeding and stroke onset further supports the hypothesis that bleeding and associated hemodynamic disturbances may act as precipitating factors for ischemic events. Due to the limited number of cases, the mechanisms outlined remain speculative, and further research is needed to validate and refine these hypotheses.

### 4.2 Diagnostic and therapeutic considerations

All patients with adenomyosis-associated stroke initially presented to neurology departments, underscoring the need for interdisciplinary recognition. Diagnosis typically requires integrating gynecological history (e.g., menorrhagia or AUB), anemia, neurological symptoms, and supportive findings such as elevated CA125/D-dimer, neuroimaging evidence of infarction or vascular stenosis, and ultrasonographic or MRI confirmation of adenomyosis. Additional risk factors, including infection and hormone replacement therapy, should be assessed, while excluding conventional stroke etiologies.

**Figure 2:**
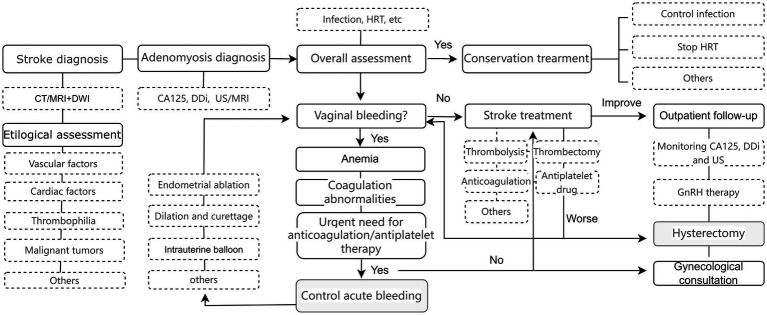
Treatment for ischemic stroke patients with adenomyosis.

The core management strategy involves identifying and removing precipitating factors (e.g., bleeding, infection, prothrombotic states), while ensuring adequate cerebral perfusion. In cases without active bleeding, standard stroke therapies such as thrombolysis, thrombectomy, and antithrombotic agents can be administered safely. However, when patients present with concomitant anemia and active menstruation, antiplatelet therapy is often contraindicated, narrowing the therapeutic window and potentially worsening outcomes. Previous studies have identified moderate to severe anemia as a predictor of poor stroke prognosis (29), underscoring the importance of rapid and effective hemostasis—a view shared by Kim et al. (30). We therefore propose a stepwise management algorithm for adenomyosis-associated ischemic stroke that integrates both neurological and gynecological decision points (Figure 2).

### 4.3 Surgical interventions: hysterectomy and alternatives

Among conservative therapies, GnRH agonists (GnRH-a) are preferred because they suppress estrogen levels, reduce bleeding, and carry a lower thrombotic risk (31, 32). In this review, 90% of medically treated patients received GnRH-a. However their benefit is temporary, as recurrence is common after discontinuation (e.g., cases (1, 7, 12, 17)), with a recurrence rate as high as 83.3% in menstruation-related cases, consistent with findings by Zhao et al. (10).

Surgical treatment was performed in 59.1% of patients, with hysterectomy being the predominant choice. This approach effectively eradicates adenomyotic lesions, resolves AUB, and normalizes CA125 and D-dimer levels. Importantly, no stroke recurrences were reported postoperatively. Arai et al. (12) recommended performing hysterectomy within 7 days of stroke onset to optimize long-term outcomes. As a definitive intervention, hysterectomy addresses both irregular uterine bleeding and the hypercoagulable state; however, it is a major procedure and poses specific challenges in the acute phase of ischemic stroke. These include the risks associated with perioperative discontinuation of antithrombotic therapy and the potential for intra-abdominal hemorrhage or thromboembolic events. Indeed, postoperative complications such as intra-abdominal bleeding (13) and pulmonary embolism (11) have been documented.

In the acute stage, when continuation of antithrombotic therapy is essential, less invasive hemostatic alternatives may be considered for patients requiring urgent bleeding control but who cannot tolerate major surgery. These include endometrial ablation, diagnostic curettage and intrauterine balloon tamponade. Although systematic evidence is limited, these procedures have been successfully applied in the management of abnormal uterine bleeding in women on anticoagulation or those with inherited bleeding disorders (33, 34), and they appear to provide satisfactory hemostasis.

### 4.4 Limitations, clinical implications, and future directions

This review is limited by the small number of published cases and the methodological shortcomings inherent to case report designs. The case reports are geographically concentrated in East Asia, which may be attributed to several factors, including regional differences in disease diagnosis and management (35, 36), the rarity of the condition, and the lack of recognition due to its interdisciplinary nature. The evidence is derived exclusively from case reports, which are subject to reporting bias, incomplete follow-up, and heterogeneity in diagnostic and therapeutic approaches. As such, current findings should be regarded as hypothesis-generating rather than definitive.

Nevertheless, several important clinical implications can be drawn. Clinicians should recognize adenomyosis-related abnormal uterine bleeding as a potential risk factor for recurrent ischemic events in women, particularly during menstruation. Early gynecological intervention and rapid hemostasis may be critical to reducing recurrence risk. Greater clinical awareness and proactive interdisciplinary management are essential for improving outcomes in this rare but serious condition. Furthermore, high-quality prospective studies are urgently needed to establish evidence-based protocols and to evaluate the safety and efficacy of minimally invasive procedures such as endometrial ablation.

## 5 Conclusion

Adenomyosis-associated abnormal uterine bleeding complicated by ischemic stroke is a rare but high-risk condition, with pathogenesis linked to anemia and a hypercoagulable state. Although current evidence is limited to case reports, the available studies suggest that conservative management carries a the available studies of recurrence, particularly in patients with menstruation-related onset. By contrast, hysterectomy as a definitive surgical intervention appears to be associated with more favorable outcomes. These findings emphasize the importance of timely hemostasis to facilitate safe antithrombotic therapy and highlight the urgent need for high-quality prospective studies to inform evidence-based clinical practice.

## Data Availability

The original contributions presented in the study are included in the article/Supplementary material, further inquiries can be directed to the corresponding author.
