# Supplementary material for: Ischemic stroke associated with adenomyosis-related abnormal uterine bleeding: a systematic review of management and outcomes
Source: Front Neurol. 2025 Dec 4;16:1698533. doi: 10.3389/fneur.2025.1698533 (PMC12711478; doi:10.3389/fneur.2025.1698533)
Supplement: Supplementary file 1 [file Data_Sheet_1.pdf]

Supplementary File S1. Detailed Search Strategy

A systematic literature search was conducted across four databases: PubMed, Web of Science, CNKI (China National Knowledge Infrastructure), and Wanfang Data. The search included publications from database inception to November 20, 2024. No language restrictions were applied. Search terms were adapted to the syntax of each database.

PubMed

("adenomyosis"[MeSH] OR adenomyosis[tiab]) AND ("ischemic stroke" OR "cerebral infarction" OR stroke[tiab])

Web of Science

Topic = ("adenomyosis") AND Topic = ("ischemic stroke" OR "cerebral infarction" OR "stroke")

CNKI and Wanfang

Subject terms and keywords used included “子宫腺肌病” or “腺肌症” AND “缺血性脑卒中” or “脑梗死” or “脑梗塞”. Both simplified and traditional Chinese expressions were included.
