# Supplementary material for: Ischemic stroke associated with adenomyosis-related abnormal uterine bleeding: a systematic review of management and outcomes
Source: Front Neurol. 2025 Dec 4;16:1698533. doi: 10.3389/fneur.2025.1698533 (PMC12711478; doi:10.3389/fneur.2025.1698533)
Supplement: Supplementary file 2 [file Table_1.pdf]

Supplementary Table S2. Risk-of-bias (JBI) summary

Case Report	Demographics described	History described	Clinical condition clear	Diagnostics described	Interventions described	Post-intervention outcomes	Adverse events reported	Clinical implications given
1.Soeda 2011(Japan)	Yes	Yes	Yes	Yes	Yes	Yes	Yes	Yes
2. Yamashiro 2012 (Japan)	Yes	Partial	Yes	Partial	Partial	Partial	Partial	Yes
3. Nishioka 2014 (Japan)	Yes	Yes	Partial	Yes	Yes	Yes	Yes	Yes
4. Hijikata 2016 (Japan)	Yes	Yes	Partial	Partial	Yes	Yes	Yes	Yes
5. Kim 2017 (Korea)	Yes	Yes	Yes	Yes	Yes	Yes	Yes	Yes
6. Uchino 2017 (Japan)	Yes	Yes	Yes	Yes	Yes	Yes	Yes	Yes
7. Aso 2018 (Japan)	Yes	Yes	Yes	Yes	Yes	Yes	Yes	Yes
8. Okazaki 2018 (Japan)	Yes	Yes	Yes	Yes	Yes	Yes	Partial	Yes
9. Yin 2018 (China)	Yes	Yes	Yes	Yes	Partial	Partial	Partial	Partial
10. Zhao 2020 (China)	Yes	Yes	Yes	Yes	Yes	Yes	Yes	Yes
11. Aiura 2021 (Japan)	Yes	Yes	Yes	Yes	Yes	Yes	Yes	Yes
12. Arai 2022 (Japan)	Yes	Yes	Yes	Yes	Yes	Yes	Yes	Yes
13. Yasuda 2022 (Japan)	Yes	Yes	Yes	Yes	Yes	Yes	Yes	Yes
14. Tamura 2022 (Japan)	Yes	Yes	Yes	Yes	Yes	Yes	Yes	Yes
15. Zhang 2022 (China)	Yes	Yes	Partial	Yes	Yes	Yes	Yes	Yes
16. Seo 2023 (Korea)	Yes	Yes	Yes	Yes	Yes	Yes	Yes	Yes

17. Morishima 2023 (Japan)	Yes	Yes	Yes	Yes	Yes	Yes	Yes	Yes
18. Chi 2024 (China)	Yes	Yes	Yes	Yes	Yes	Yes	Yes	Yes
