# Supplementary material for: Ischemic stroke associated with adenomyosis-related abnormal uterine bleeding: a systematic review of management and outcomes
Source: Front Neurol. 2025 Dec 4;16:1698533. doi: 10.3389/fneur.2025.1698533 (PMC12711478; doi:10.3389/fneur.2025.1698533)
Supplement: Supplementary file 3 [file Table_2.pdf]

Supplementary Table S3. Clinical characteristics and outcomes of 24 patients with adenomyosis-associated ischemic stroke.

Source references correspond to the main text citations [1-18].

Case	Author, Year	Age	Country	Primary Department	Clinical Presentation	Menstrual Period	CA-125 (U/mL)	D-dimer (μg/mL)	Hb (g/L)	NBTE	Extracerebral Infarction	Treatment for Cerebral Infarction	Treatment for Adenomyosis	Hysterectomy	Recurrence	Reference Number
1	Soeda S, 2011	50	Japan	Neurology	Hemianopsia	Yes	1519	95.7	83	Yes	No	No anticoagulation	GnRH agonist	Yes	Yes→No	[1]
2	Yamashiro K, 2012	45	Japan	Neurology	Hemiparesis, impaired consciousness	No	159	1.1	84	No	Left fingers, BCT, LSA	UFH→ Antiplatelet	GnRH agonist	No	-	[2]
3	Yamashiro K, 2012	44	Japan	Neurology	Hemiplegia	No	-	-	70	No	Kidney	UFH→Warfarin	GnRH agonist	No	-	[2]
4	Yamashiro K, 2012	50	Japan	Neurology	Hand weakness	Yes	42.6	0.57	69	No	No	Aspirin	GnRH agonist	No	-	[2]
5	Yamashiro K, 2012	42	Japan	Neurology	Aphasia	Yes	1750	6	86	No	No	1st: Antiplatelet, 2nd: UFH→ Warfarin	GnRH agonist	No	-	[2]
6	Nishioka K, 2014	47	Japan	Neurology	Epilepsy	-	784.6	6.3	76	No	No	UFH→Warfarin	-	Yes	No	[3]
7	Hijikata N, 2016	59	Japan	Neurology	Limb weakness	-	334.8	7	-	Yes	No	UFH	Discontinued HRT	No	No	[4]
8	Kim B, 2017	49	Korea	Neurology	Dysarthria, sensory change	No	379	3.99	99	Yes	No	LMWH→ Warfarin	-	Yes	No	[5]
9	Uchino K, 2017	48	Japan	Neurology	Hemiparesis, aphasia	No	901	1.9	85	Yes	No	UFH→Warfarin	-	Yes	No	[6]

10	Aso Y, 2018	44	Japan	Neurology	Hand weakness, gait disturbance	Yes	2115	17	103	No	Spleen	UFH→ Rivaroxaban/Warfarin	GnRH agonist	Yes	Yes→No	[7]
11	Okazaki K, 2018	42	Japan	Neurology	Hemiparesis， aphasia	No	395	1.4	-	No	No	Warfarin	-	No	No	[8]
12	Okazaki K, 2018	50	Japan	Neurology	Hemiparesis， aphasia	No	143	3.7	-	No	No	Rivaroxaban	-	No	No	[8]
13	Yin X, 2018	34	China	Neurology	Vertigo	Yes	937.1	1.05	134	No	No	-	-	-	-	[9]
14	Yin X, 2018	37	China	Neurology	Limb weakness	Yes	735.7	2.34	108	No	No	-	-	-	-	[9]
15	Yin X, 2018	46	China	Neurology	Hemiplegia	Yes	546.5	12	121	No	No	-	-	Yes	No	[9]
16	Zhao Y, 2020	34	China	Neurology	Fever, limb weakness	Yes	937.7	27.4	112	No	No	LMWH→ Clopidogrel	-	No	No	[10]
17	Aiura R, 2021	48	Japan	Neurology	Fever, impaired consciousness	Yes	3536.2	79.3	82	No	Bilateral kidneys	UFH→Edoxaban, endovascular thrombectomy	-	Yes	No	[11]
18	Arai N, 2022	50	Japan	Neurology	Hemianopsia	Yes	999	6.4	92	No	No	UFH→Apixaban	GnRH agonist	Yes	Yes→No	[12]
19	Yasuda M, 2022	47	Japan	Neurology	Hand weakness, aphasia	Yes	90.3	3.8	113	No	Kidney	UFH→Edoxaban	-	Yes	Yes→No	[13]
20	Tamura M, 2022	46	Japan	Neurology	Hemiparesis, aphasia	Yes	1477	7.4	96	No	Left ICA, right leg, lung	Aspirin→UFH→ Rivaroxaban	-	Yes	No	[14]
21	Zhang A, 2022	38	China	Neurology	Limb weakness	-	14.07	0.26	61	No	No	Aspirin→ Revascularization surgery	-	Yes	No	[15]
22	Seo J, 2023	47	Korea	Neurology	Vertigo, impaired	Yes	48	13.66	34	Yes	No	Warfarin	Medroxyprogesterone	Yes	No	[16]

					consciousness											
23	Morishima Y, 2023	42	Japan	Neurology	Ataxia	Yes	576	9.7	132	No	Bilateral kidneys	UFH→Edoxaban	GnRH agonist	Yes	Yes→No	[17]
24	Chi B, 2024	46	China	Neurology	Headaches, altered consciousness	Yes	253	11	23	No	No	UFH	GnRH agonist	No	-	[18]

Abbreviations:
CA-125: Cancer Antigen 125, D&C: Dilation and Curettage, GnRH: Gonadotropin-Releasing Hormone, Hb: Hemoglobin, HRT: Hormone Replacement Therapy, BCT: Brachiocephalic Trunk, ICA: Internal Carotid Artery, LSA: Left Subclavian Artery, LMWH: Low Molecular Weight Heparin, NBTE: Nonbacterial Thrombotic Endocarditis, PE: Pulmonary Embolism, UFH: Unfractionated Heparin

Notes:
Normal reference ranges: CA-125 <35 U/mL; D-dimer <0.5 μg/mL; Hemoglobin 12-16 g/dL (female)
"Yes→No" indicates initial recurrence followed by resolution after definitive treatment
"-" indicates data not available in the original report
